# Is Neck Circumference Related to Other Anthropometric Measurements and Biochemical Parameters in Type 2 Diabetes?

**DOI:** 10.7759/cureus.30750

**Published:** 2022-10-27

**Authors:** Hulya Kamarli Altun, Gulen Suna

**Affiliations:** 1 Department of Nutrition and Dietetics, Akdeniz University, Faculty of Health Sciences, Antalya, TUR

**Keywords:** type 2 diabetes, anthropometric measurements, body mass index, hip circumference, neck circumference, waist circumference

## Abstract

Background

Obesity is one of the leading risk factors for developing type 2 diabetes mellitus (T2DM). Body mass index (BMI), waist circumference, and waist/hip ratio are anthropometric measurements used to diagnose obesity. In recent years, neck circumference, one of the anthropometric indicators used in assessing obesity, has come to the fore. This study investigates the relationship between neck circumference and other anthropometric measurements and specific biochemical parameters in T2DM.

Methods

Four hundred sixty-four individuals with type 2 diabetes were included in the study. Subjects’ body weight, height, and other anthropometric measurements like circumferences of the waist, hip, and neck were measured. BMI, waist/hip, and waist/height ratio were calculated. The biochemical tests of the subjects in the previous month from the study were accessed from the hospital information system. The relationship between anthropometric measurements and biochemical parameters with neck circumference was evaluated.

Results

The mean age of the subjects was 54.6±8.51 years. 56.2% were female and 43.8% were male, and the time from T2DM diagnosis was 9.9±7.49 years. Most male subjects were overweight (49.8%), and approximately one-third of the women were first-degree obese (33.0%). Body weight, BMI, waist, hip, and neck circumferences, and waist/hip and waist/height ratios were significantly higher in both male and female subjects in the neck circumference high-risk group than those in the low-risk group (p<0.05). In terms of biochemical parameters, fasting blood glucose and HbA1c values of men with neck circumference at risk were significantly higher than those in the low-risk group. Neck circumference measurements of the participants were positively correlated with body weight (r=0.543; p<0.001), height (r=0.260; p<0.001), waist circumference (r=0.562; p<0.001), hip circumference (r=0.293; p<0.001), BMI (r=0.366; p<0.001), waist/hip ratio (r=0.428, p<0.001), and waist/height ratio (r=0.393, p<0.001). Neck circumference had a low positive correlation with fasting blood glucose (r=0.165; p<0.001), HbA1c (r=0.281; p<0.001), and triglyceride (r=0.231; p<0.001) and a negative relationship with low-density lipoprotein cholesterol (r=-0.118; p=0.001). When the relationship between neck circumference and biochemical parameters was examined, it was seen that this correlation was only in men.

Conclusion

Neck circumference measurement is a simple and reliable method and is not affected by external factors. It correlates with other anthropometric measurements and can be used as a good indicator of the distribution of upper subcutaneous adipose tissue in T2DM. However, more studies with larger samples are needed on this subject.

## Introduction

Diabetes mellitus is an important public health problem with an increasing prevalence [1]. The International Diabetes Federation (IDF) estimates that 537 million adults living with diabetes existed worldwide in 2021, and it is projected to reach 783 million by 2045. Additionally, type 2 diabetes poses a risk to 541 million persons who do not currently have the disease [2].

Obesity, also known as excessive fat accumulation in the body, is one of the leading risk factors for developing type 2 diabetes mellitus (T2DM) [3,4]. About 80-90% of patients with T2DM are overweight or obese [4]. Obesity is not only a cause of insulin resistance but also a growing health problem in its own right [5]. Therefore, early diagnosis of obesity and taking necessary precautions are vital. Various methods and indicators are used to measure excessive fat accumulation in the body.

Although body mass index (BMI) is a relatively simple and well-known method for diagnosing obesity, it is insufficient for evaluating the amount and distribution of fat in the body. Other commonly used methods are waist circumference (WC), waist/hip ratio (WHR), waist/height ratio (WHtR), and skinfold thickness [6]. Some studies state that the distribution of body fat may be more effective in the formation of diseases than the amount of adipose tissue in the body [7,8]. The development of chronic illnesses like T2DM and coronary artery disease is influenced by abdominal adipose tissue [9]. Unfortunately, dual-energy X-ray absorptiometry (DEXA), tomography, and bioelectrical impedance analysis (BIA) are costly methods used to determine body fat distribution. Therefore, the use of these methods is limited [9,10]. Instead of these reliable but costly methods with low applicability, WC measurement is widely used as an indicator of abdominal fat. Waist circumference measurement is considered a more specific metabolic risk indicator than BMI, as it differentiates between visceral fat and lean body mass in the abdomen [11]. In recent years, the use of neck circumference (NC) has come to the fore as a stable index to determine the distribution of subcutaneous adipose tissue as an indicator of abdominal obesity [12]. WC measurement is affected by distention and respiration; however, the NC measurement is an easy-to-apply anthropometric measurement that remains unaffected when measured throughout the day. It is also associated with risk factors such as hyperlipidemia, hyperglycemia, blood pressure, insulin resistance, and obstructive sleep apnea, independent of BMI and WC [11]. 

Obesity can lead to insulin resistance and worsen diabetes-specific biochemical parameters as well as a blood lipid profile. A study conducted in Turkey showed that NC, which is an indicator of obesity, was associated with many parameters; however, it was not related to fasting blood glucose (FBG), glycated hemoglobin (HbA1c), and low-density lipoprotein cholesterol (LDL-C) [13]. 

This study aimed to evaluate the correlation between NC and other anthropometric measurements and specific metabolic parameters in individuals with T2DM.

## Materials and methods

Study design

This cross-sectional study included 464 adults (ages 18-65) with T2DM who applied to Akdeniz University Hospital Endocrinology and Metabolic Diseases outpatient clinic between March 2022 and July 2022. Patients younger than 18 and older than 65; who had type 1 diabetes, thyroid diseases, and an amputated limb; were wheelchair dependent and unable to stand up; suffered from osteoporosis or disorders that may affect posture; were pregnant or lactating; and those who did not complete the questionnaire were excluded from the study. The study was approved by the Akdeniz University Faculty of Medicine Clinical Research Ethics Committee. This study was accomplished by following the guidelines in the Declaration of Helsinki, and all patients who participated in the study gave informed consent.

Data collection

The subjects were questioned in terms of demographic features and health information, such as diseases, time from T2DM diagnosis, and treatments used. The classification of glucose-lowering agents that effects weight change has been made according to the American Diabetes Association's Standards of Medical Care in Diabetes-2022 [14]. The patients' FBG, HbA1c, direct LDL-C, and triglyceride (TG) values​ in the previous month from the study were accessed from the hospital information system. Body weight, height, NC, WC, and hip circumference (HC) measurements were taken. To ensure accuracy, all anthropometric measurements were obtained by the same dietitian in triplicate using a rigid measuring tape with an accuracy of 0.1 cm. WC was measured with the subject standing in the Frankfort plane, hands and arms at both sides, feet close to each other, by calculating the circumference passing through the middle of the distance between the lowest rib and the crista iliac superior with a rigid measuring tape. HC was measured at the largest part of the hip with a rigid measuring tape standing next to the subject with their arms at the sides and legs together in the Frankfort plane [15]. NC was also measured from the larynx inferior with a measuring tape while the individual was standing in the Frankfort plane. The individual was asked not to hold their breath during the process, and the measurement was taken as soon as the exhalation was finished [15].

The BMI was calculated using the formula of body weight (kg)/height^2^ (m^2^) and classified by World Health Organization's recommendation. WC, NC, WHR, and WHtR were assessed by classifying them based on risk values. WC was classified as a risk when it was 80-88 cm for women and 94-102 cm for men and as high risk when it was ≥88 cm for women and ≥102 cm for men. Moreover, NC was evaluated as a risk when it was ≥34 cm for women and ≥37 cm for men, just as the WHtR when it was ≥0.5. On the other hand, WHR was classified as high risk when it was ≥0.85 for women and ≥0.90 for men [16].

Statistical analysis

The study data were evaluated using the SPSS 23.0 statistical software (IBM Inc., Armonk, New York) [17]. Qualitative data were presented in numbers (n) and percentages (%), and Pearson chi-square and Fisher-Freeman-Hamilton tests were used to analyze categorical variables. Quantitative data were presented as mean and standard deviation. In case of differences between groups in cross-table analyses, pairwise comparisons were made with a Bonferroni-adjusted ratio comparison. Compliance of quantitative data with normal distribution was analyzed with the Kolmogorov-Smirnov test. Since the variables did not have a normal distribution, the Mann-Whitney U test was used for comparison. Spearman correlation analysis was employed to investigate the relations between neck circumference, other anthropometric measurements, and specific biochemical parameters. Correlation analysis was also performed separately based on gender-specific neck circumference risk values. Significance was accepted as p<0.05 for all statistical analyses.

## Results

The socio-demographic characteristics of the participants are shown in Table 1. The mean age of the participants was 54.6±8.51 years. 56.2% were female, and the time from T2DM diagnosis was 9.9±7.49 years. Approximately 73.5% of patients had a disease other than diabetes, and the most common were hypertension (42%) and cardiovascular diseases (38.1%). It was observed that the majority of the subjects used lipid-lowering agents (76.3%), and the most commonly used drugs in diabetes treatment were metformin (47.2%) and insulin (37.5%). When the effects of glucose-lowering drugs on body weight were evaluated, it was determined that the participants mainly used drugs that did not affect body weight (40.3%) and caused weight loss (39.2%).

**Table 1:** General characteristics of the participants SGLT2: sodium-glucose cotransporter-2, DPP-4: dipeptidyl peptidase-4, GLP-1 RAs: glucagon-like peptide-1 receptor agonists, DM: diabetes mellitus * The total number is greater than n due to participants having more than one disease and using more than one glucose-lowering agent ^a^Fisher-Freeman-Hamilton test, ^b^Pearson chi-square, p<0.05

Variables	Male (n=203)	Female (n=261)	p-value
	n (%)	n (%)	
Marital status			
Married	186 (91.6)	224 (85.8)	0.581^a^
Single	17 (8.4)	37 (14.2)	
Education status			
Illiterate	3 (1.5)	23 (8.8)	
Literate	39 (19.2)	69 (26.4)	
Primary school	51 (25.1)	66 (25.3)	0.002^b^
Secondary school	49 (24.1)	41 (15.7)	
High school	49 (24.1)	53 (20.3)	
Bachelor/ postgraduate degree	12 (5.9)	9 (3.4)	
Working status			
Employee	71 (35.0)	37 (14.2)	
Unemployed	102 (50.2)	219 (83.9)	<0.001^b^
Retired	30 (14.8)	5 (1.9)	
Having chronic disease except DM			
Yes	145 (71.4)	196 (75.1)	0.397^a^
No	58 (28.6)	65 (24.9)	
Comorbities*			
Hypertension (n=195)	75 (36.9)	120 (46.0)	0.058^a^
Cardiovascular diseases (n=177)	84 (41.4)	93 (35.6)	0.212^a^
Kidney diseases (n=30)	12 (5.9)	18 (6.9)	0.708^a^
Gastrointestinal system diseases (n=41)	11 (5.4)	30 (11.5)	0.031^a^
Pulmonary diseases (n=31)	12 (5.9)	19 (7.3)	0.581^a^
Neurologic diseases (n=18)	2 (1.0)	16 (6.1)	0.006^a^
Other (n=45)	9 (4.4)	36 (13.8)	0.596^a^
Time from T2DM diagnosis			
0-10 years	124 (61.1)	175 (67.0)	
11-20 years	61 (30.0)	66 (25.3)	0.411^b^
>21 years	18 (8.9)	20 (7.7)	
Lipid-lowering and glucose-lowering agents use status*			
Lipid-lowering agents (n=354)	166 (81.8)	188 (72.0)	0.016^a^
Metformin (n=219)	80 (39.4)	139 (53.3)	0.004^a^
Sulfonylureas (n=64)	33 (16.3)	31 (11.9)	0.178^a^
Glitazone (n=26)	11 (5.4)	15 (5.7)	1.000^a^
Alpha-glucosidase inhibitors (n=7)	2 (1.0)	5 (1.9)	0.475^a^
SGLT2 Inhibitors (n=88)	40 (19.7)	48 (18.4)	0.722^a^
DPP-4 Inhibitors (n=69)	26 (12.8)	43 (16.5)	0.295^a^
GLP-1 RAs (n=9)	2 (1.0)	7 (2.7)	0.310^a^
Insulin (n=174)	87 (42.9)	87 (33.3)	0.042^a^
Basal Insulin (n=56)	26 (29.9)	30 (34.5)	0.627^a^
Basal and Bolus Insulin (n=118)	61 (70.1)	57 (65.5)	
Effect of glucose-lowering agents used on body weight change *			
Neutral (n=187)	73 (36.0)	114 (43.8)	0.298^b^
Loss (n=47)	20 (9.9)	27 (10.3)	
Gain (n=182)	89 (43.8)	93 (35.6)	
Loss and gain (n=48)	21 (10.3)	27 (10.3)	

Participants' anthropometric measurements and biochemical parameters' data are shown in Table 2. The mean body weight, WC, HC, NC, and BMI values ​​of the individuals were 80.2±14.08 kg, 102.2±12.99 cm, 107.4±11.68 cm, 37.7±3.63 cm, and 29.8±5.37 kg/m^2^, respectively. The BMI, WC and HC measurements, and WHtR of women in the study were significantly higher than those of men (p<0.05). The mean values of FBG, HbA1c, TG, and direct LDL-C from biochemical parameters were 161.5±63.17 mg/dL, 7.55±1.94%, 174.4±92.65 mg/dL, and 119.0±34.97mg/dL, respectively. Of the biochemical parameters, only FBG was significantly lower in women than in men (p=0.009). Most males were overweight (49.8%), and approximately one-third of the women were first-degree obese (33.0%). Most participants had their WC in the high-risk group, and the number of women at increased risk was higher than men (F: 88.1%, M: 42.9%; p<0.001). Considering the risk group of the subjects based on their WHR, WHtR, and NC, most of them were at high risk. The number of women at risk, according to WHR and NC, was greater than men (p<0.05). 

**Table 2:** Anthropometric measurements and biochemical parameters of the participants FBG: fasting blood glucose, TG: triglyceride, LDL-C: low-density lipoprotein cholesterol, WC: waist circumference, HC: hip circumference, NC: neck circumference, WHR: waist/hip ratio, WHtR: waist/height ratio, BMI: body mass index Waist circumference classification: normal M<94cm, F<80cm; risk M=94-102 cm, F=80-88cm; high-risk M≥102cm, F≥88cm Waist/hip ratio classification: low-risk M<0.90, F<0.85; high-risk M≥0.90; F≥0.85. Waist/height ratio classification: normal <0.5; high-risk ≥0.5 Neck circumference classification: normal M<37cm, F<34cm; high-risk M≥37cm, F≥34 cm ^a^Mann-Whitney U test, ^b ^Pearson Chi-square, ^c^Fisher-Freeman-Hamilton test, p<0.05

Variables	Total (n=464)	Male (n=203)	Female (n=261)	p-value
	X±SD	X±SD	X±SD	
Anthropometric measurements				
Body weight (kg)	80.2±14.08	82.3±14.34	78.6±13.68	0.008^a^
Height (cm)	164.4±9.19	171.0±7.33	159.2±6.90	<0.001^a^
BMI (kg/m^2^)	29.8±5.37	28.1±4.13	31.1±5.84	<0.001^a^
WC (cm)	102.2±12.99	101.0±12.74	103.0±13.14	0.028^a^
HC (cm)	107.4±11.68	103.9±9.27	110.1±12.62	<0.001^a^
NC (cm)	37.7±3.63	39.4±3.69	36.5±3.05	<0.001^a^
WHR	0.95±0.09	0.97±0.09	0.94±0.09	<0.001^a^
WHtR	0.62±0.09	0.59±0.07	0.65±0.09	<0.001^a^
Biochemical parameters				
FBG (mg/dL)	161.5±63.17	170.5±67.89	154.5±58.42	0.009^a^
HbA1c (%)	7.55±1.94	7.66±2.17	7.46±1.74	0.772
TG (mg/dL)	174.4±92.65	192.0±114.75	161.6±69.92	0.065
LDL-C (mg/dL)	119.0±34.97	119.6±33.82	118.5±35.89	0.369
	Total (n=464)	Male (n=203)	Female (n=261)	p-value
	n (%)	n (%)	n (%)	
BMI classification				
18.5-24.99	86 (18.5)	46 (22.7)	40 (15.3)	<0.001^b^
25.0-29.99	176 (37.9)	101 (49.8)	75 (28.7)	
30.0-34.99	125 (26.9)	39 (19.2)	86 (33.0)	
35.0-39.99	56 (12.1)	16 (7.9)	40 (15.3)	
>40.0	21 (4.5)	1 (0.5)	20 (7.7)	
WC classification				
Normal	85 (18.3)	70 (34.5)	15 (5.7)	<0.001^b^
Risk	62 (13.4)	46 (22.7)	16 (6.1)	
High-risk	317 (68.3)	87 (42.9)	230 (88.1)	
WHR classification				
Low-risk	85 (18.3)	48 (23.6)	37 (14.2)	0.011^c^
High-risk	379 (81.7)	155 (76.4)	224 (85.8)	
WHtR classification				
Normal	33 (7.1)	18 (8.9)	15 (5.7)	0.207^c^
High-risk	431 (92.9)	185 (91.1)	246 (94.3)	
NC classification				
Normal	94 (20.3)	54 (26.6)	40 (15.3)	0.003^c^
High-risk	370 (79.7)	129 (73.4)	221 (84.7)	

Table 3 demonstrates the evaluation of the anthropometric measurements, biochemical parameters, and use of lipid and glucose-lowering agents of male and female subjects based on the high-risk and low-risk groups of NC. Body weight, BMI, WC, HC, NC, WHR, and WHtR were significantly more elevated in both male and female subjects in the high-risk group than in the low-risk group (p<0.05). FBG and HbA1c values of men with NC at risk were significantly higher than those in the low-risk group, while direct LDL-C was significantly lower in the high-risk group (p<0.05). In contrast, no significant difference was found in women. When glucose and lipid-lowering agents were evaluated, it was observed that insulin use was significantly higher in men only in the high-risk group of NC than in the low-risk group (p<0.001). Likewise, it was found that the use of glucose-lowering agents leading to body weight gain was higher in men with a high-risk group of NC (p=0.001).

**Table 3:** Evaluation of the anthropometric measurements, biochemical parameters, and lipid and glucose-lowering agents in relation to the neck circumference of the participants according to the low and high-risk groups FBG: fasting blood glucose, TG: triglyceride, LDL-C: low-density lipoprotein cholesterol, SGLT2: sodium-glucose cotransporter-2, DPP-4: dipeptidyl peptidase-4, GLP-1 RAs: glucagon-like peptide-1 receptor agonists, WC: waist circumference, HC: hip circumference, NC: neck circumference, WHR: waist/hip ratio, WHtR: waist/height ratio, BMI: body mass index ^a^Mann-Whitney U test, ^b^Pearson Chi-square, p<0.05

Variables		Male (n=201)		p-value	Female (n=261)		p-value
		NC- low risk (n=54)	NC- high risk (n=149)		NC- low risk (n=40)	NC- high risk (n=221)	
		X±SD	X±SD		X±SD	X±SD	
Anthropometric measurements							
Body weight (kg)		72.9±7.94	85.8±14.63	<0.001^a^	66.3±9.81	80.8±13.10	<0.001^a^
Height (cm)		169.4±6.68	171.6±7.48	0.213^a^	160.5±6.28	159.0±6.99	0.175^a^
BMI (kg/m^2^)		24.4±1.96	29.1±4.27	<0.001^a^	25.9±4.49	32.1±5.55	<0.001^a^
WC (cm)		88.8±5.43	105.4±11.70	<0.001^a^	88.7±13.07	105.6±11.34	<0.001^a^
HC (cm)		100.3±9.33	105.3±8.92	<0.001^a^	100.6±11.04	111.9±12.13	<0.001^a^
NC (cm)		34.8±1.22	41.0±2.79	<0.001^a^	32.2±1.03	37.3±2.60	<0.001^a^
WHR		0.89±0.06	1.00±0.07	<0.001^a^	0.88±0.08	0.95±0.09	<0.001^a^
WHtR		0.53±0.04	0.62±0.07	<0.001^a^	0.56±0.09	0.67±0.08	<0.001^a^
Biochemical parameters							
FBG (mg/dL)		146.2±39.86	179.3±73.69	0.013^a^	143.8±59.21	156.4±58.20	0.171^a^
HbA1c (%)		6.3±1.88	8.16±2.05	<0.001^a^	7.3±2.03	7.5±1.69	0.268^a^
TG (mg/dL)		143.4±46.33	196.4±118.18	0.193^a^	138.0±53.93	164.0±71.10	0.195^a^
LDL-C (mg/dL)		132.7±27.38	114.9±34.76	0.001^a^	121.7±35.31	117.9±36.05	0.426^a^
Lipid-lowering and glucose-lowering agents		n (%)	n (%)		n (%)	n (%)	
Lipid-lowering drugs	Yes	45 (83.3)	121 (81.2)	0.838^b^	28 (70.0)	160 (72.4)	0.848^b^
	No	9 (16.7)	28 (18.8)		12 (30.0)	61 (27.6)	
Metformin	Yes	18 (33.3)	62 (41.6)	0.331^b^	23 (57.5)	116 (52.5)	0.608^b^
	No	36 (66.7)	87 (58.4)		17 (42.5)	105 (47.5)	
Insulin	Yes	5 (9.3)	82 (55.0)	<0.001^b^	10 (25.0)	77 (34.8)	0.276^b^
	No	49 (90.7)	67 (45.0)		30 (75.0)	144 (65.2)	
SGLT2 inhibitors	Yes	6 (11.1)	34 (22.8)	0.074^b^	4 (10.0)	44 (19.9)	0.183^b^
	No	48 (88.9)	115 (77.2)		36 (90.0)	177 (80.1)	
DPP-4 inhibitors	Yes	4 (7.4)	22 (14.8)	0.235^b^	10 (25.0)	33 (14.9)	0.161^b^
	No	50 (92.6)	127 (85.2)		30 (75.0)	188 (85.1)	
Sulfonylureas	Yes	5 (9.3)	28 (18.8)	0.132^b^	3 (7.5)	28 (12.7)	0.437^b^
	No	49 (90.7)	121 (81.2)		37 (92.5)	193 (87.3)	
Glitazone	Yes	1 (1.9)	10 (6.7)	0.294^b^	2 (5.0)	13 (5.9)	1.000^b^
	No	53 (98.1)	139 (93.3)		38 (95.0)	208 (94.1)	
GLP-1 RAs	Yes	-	2 (1.3)	1.000^b^	-	7 (3.2)	0.600^b^
	No	54 (100.0)	147 (98.7)		40 (100.0)	214 (96.8)	
Alfa-glucosidase inhibitors	Yes	1 (1.9)	1 (0.7)	0.462^b^	-	5 (2.3)	1.000^b^
	No	53 (98.1)	148 (99.3)		40 (100.0)	216 (97.7)	
Effect of glucose-lowering agents used on body weight change							
Neutral		39 (72.2)	3 (22.8)	0.001^b^	21 (52.5)	93 (42.1)	0.123^b^
Loss		4 (7.4)	16 (10.7)		4 (10.0)	23 (10.4)	
Gain		9 (16.7)	80 (53.7)		15 (37.5)	78 (35.3)	
Loss and gain		2 (3.7)	19 (12.8)		-	27 (12.2)	

Table 4 includes the correlation between NC, other anthropometric measurements, and biochemical parameters. In male with T2DM, a moderately significant positive correlation was found between the measurement of NC and body weight, BMI, HC, and WHR (respectively; r=0.532, r=0.568, r=0.425, and r=0.611; p<0.001), and a highly positive relationship with WC (r=0.752; p<0.001) and WHtR (r=0.723; p<0.001). Alike in female subjects, NC was significant positively correlated with body weight, BMI, WC, HC, and WHtR (respectively; r=0.578, r=0.579, r=0.584, r=0.488, and r=0.545; p<0.001) on a moderate level and with WHR (r=0.224; p<0.001) on a low level. Only males with T2DM of the biochemical parameters NC had a significantly positive moderate correlation with HbA1c (r=0.491; p<0.001), a positively low correlation with FBG (r=0.197; p=0.005), and a negatively low correlation with LDL-C (r=-0.247; p<0.001).

**Table 4:** The correlation between neck circumference and other anthropometric measurements and biochemical parameters in participants *FBG: fasting blood glucose, TG: triglyceride, LDL-C: low-density lipoprotein cholesterol Spearman correlation analysis *p<0.05; **p<0.01

Variables	Neck circumference					
	Total		Male		Female	
	r	p-value	r	p-value	r	p-value
Body weight	0.543**	<0.001	0.532**	<0.001	0.578**	<0.001
Height	0.260**	<0.001	0.032	0.650	-0.098	0.115
Body mass index	0.366**	<0.001	0.568**	<0.001	0.579**	<0.001
Waist circumference	0.562**	<0.001	0.752**	<0.001	0.584**	<0.001
Hip circumference	0.293**	<0.001	0.425**	<0.001	0.488**	<0.001
Waist/hip ratio	0.428**	<0.001	0.611**	<0.001	0.224**	<0.001
Waist/height ratio	0.393**	<0.001	0.723**	<0.001	0.545**	<0.001
FBG	0.165**	<0.001	0.197**	0.005	0.084	0.178
HbA1c	0.281**	<0.001	0.491**	<0.001	0.094	0.129
TG	0.231**	<0.001	0.165	0.088	0.106	0.200
LDL-C	-0.118*	0.011	-0.247**	<0.001	-0.065	0.296

## Discussion

Obesity, especially abdominal obesity, is one of the major risk factors for T2DM and its complications. Healthcare professionals often prefer BMI as it is a simple and practical method. However, BMI assessing excess body fat mass to reflect body composition and visceral obesity is insufficient [18,19]. NC, which has been used frequently in recent years, is a fast, non-invasive, and inexpensive alternative anthropometric measurement. It indicates the difference between normal and abnormal fat accumulation and upper subcutaneous adipose tissue distribution and does not change during the day [19,20].

Studies on NC generally suggest a relationship between NC and obesity, metabolic syndrome, coronary diseases, and sleep apnea or the risk of these diseases [18,21-23]. However, the studies investigating the association of NC with diabetes and its metabolic parameters are quite limited [13,20,24,25]. In a limited number of previous studies on people living with diabetes, no study was found that evaluated neck circumference and waist/height ratio, which is accepted as an indicator of abdominal obesity. Therefore, this study evaluated the relationship between NC and other anthropometric measurements and biochemical parameters in T2DM. As a result of the study, positive correlation was found between NC and body weight (r=0.543; p<0.001), height (r=0.260; p<0.001), WC (r=0.562; p<0.001), HC (r=0.293; p<0.001), BMI (r=0.366; p<0.001), WHR (r=0.428; p<0.001), and WHtR (r=0.393; p<0.001). When the relationship between biochemical parameters and NC was examined, a low positive correlation was found between NC and FBG (r=0.165; p<0.001), HbA1c (r=0.281; p<0.001), and TG (r=0.231; p<0.001) and a negative correlation with LDL-C (-0.118; p<0.001) in the total study sample.

A study on the correlation between NC and other anthropometric measurements in overweight and obese individuals showed a positive, strong correlation between NC and body weight, BMI, WC, HC, and WHR in both men and women and that this correlation was particularly stronger in men [21]. Another study conducted on 81 morbidly obese individuals found a statistically significant relationship between NC and WC (r=0.38; p<0.001); however, unlike other studies, it did not find any relationship with BMI (r=0.06; p=0.60) [22]. A study by Dominguez et al. on 9218 individuals from eight Latin American countries suggested a moderate positive correlation between NC and WC, and NC and BMI (respectively; r=0.64, r=0.51) in overweight individuals. The study reported a moderate correlation between NC and WC (r=0.69) in men and a strong correlation (r=0.70) in women [11].

Another study on 1206 non-diabetic people found a significant strong relationship between NC, WC, and BMI (respectively, r=0.64 and r=0.66; p<0.001) and a significant moderate relationship between NC and body fat percentage (r=0.45; p<0.001) [26]. A study done in Turkey reported a significantly moderate positive correlation between NC and body weight, WC, HC, and BMI in male subjects (respectively; r=0.576, r=0.593, r=0.568, and r=0.587; p=0.000). However, in female subjects, there was a significantly strong correlation between NC and body weight (r=0.702; p=0.000), and there were moderate correlations between NC, WC, HC, and BMI (respectively; r=0.667, r=0.617, and r=0.688; p=0.000) [27]. 

One of the studies conducted in Turkey on the correlation between NC and other anthropometric measurements in patients with T2DM suggested a statistically significant positive relationship between the NC and BMI in both genders (p<0.001) [13]. However, another study reported a moderate positive correlation between NC and WC in women (r=0.647; p=0.001) and in men (r=0.652; p=0.001) [24]. Aswathappa et al. reported that NC was positively correlated with BMI (r=0.668; p=0.001), WC (r=0.773; p=0.001) and WHR (r=0.484; p=0.001) in those with T2DM but not with body weight and hip circumference [20]. This study found a significant correlation between NC and body weight, BMI, WC, and WHR in both men and women, like the results of other studies in individuals with and without T2DM. Contrary to some studies in the literature, this study found a positive relationship between neck and hip circumferences, albeit at a low level. Also, in our study, the relationship between NC and HC may be associated with the fact that most of our subjects were female, gynoid-type obesity is frequent in women, and most of the study sample was overweight and obese. Unlike studies on NC in the literature, this study also evaluated the WHtR and found a relationship between NC and WHtR due to visceral obesity, a critical risk factor for diabetes.

A study by Assyov et al. conducted with 255 obese people, which investigated the relationship between NC and diabetes-specific biochemical parameters and where more than half of the subjects had T2DM and prediabetes, suggested a significant relationship between NC and HbA1c (F: r=0.242, M: r=0.320; p<0.001) and TG (F: r=0.278, M: r=0.309; p<0.001) in both genders, but not found a correlation between LDL-C [18]. In their study on morbidly obese patients, Scovronec et al. found a negative correlation between NC and LDL-C, and high-density lipoprotein cholesterol but not between FBG and HbA1c [22]. The study by Joshipura et al. showed that in non-diabetic individuals, a significant weak correlations were present between FBG (r=0.10; p<0.05), HbA1c (r=0.28; p<0.001) and TG (r=0.12; p<0.05) [26].

Similarly, a meta-analysis of observational studies, which included healthy individuals, suggested a significant positive relationship between NC and FBG, and nine studies found a relationship between NC and HbA1c [28]. Another meta-analysis, which includes 33 observational studies on healthy and obese, overweight, or dyslipidemic subjects investigating the correlation between NC and lipid profile, stated a correlation between NC and high-density lipoprotein cholesterol, and, in men, a positive relationship between LDL-C and TG [29]. One of the studies conducted in Turkey, which evaluated NC and specific biochemical parameters in people with T2DM, found a positive correlation (p=0.02) between NC and TG in both men and women, but not between HbA1c, FBG, and LDL-C [13]. In another study, there was a relationship between NC and TG only in men (r=0.184; p=0.036), but no correlation was found with FBG [24]. This study represented a positive correlation between NC, FBG, and HbA1c, in male subjects with T2DM. Unlike the studies on T2DM, a negative relationship between NC and LDL-C was found, such as in investigations by Assyov et al. and Scovronec et al. [18,22]. Depending on the increase in body weight, the thickening of the NC and deterioration in glycemic values ​​are expected outcomes. The significant positive correlation between NC and FBG and HbA1c in male participants of this study may be due to men's NC values, and the risk of android obesity is high. The fact that men in the high-risk group of NC mostly use insulin as a glucose-lowering agent also explains this situation. Dyslipidemia and cardiovascular diseases are common in males living with diabetes caused by body fat accumulation. The mean age of the patients with T2DM was 54.6±8.51 years, and considering that the most common diseases other than diabetes were hypertension (42%) and cardiovascular diseases (38.1%), they had at least one risk factor for cardiovascular disease. Cardiovascular diseases, one of the most common macrovascular complications in poorly controlled T2DM, are the leading reasons for morbidity and mortality [30]. Therefore, apart from the medical treatment of T2DM, additional medical therapies are usually administered to improve dyslipidemia and prevent or delay the development of atherosclerotic heart diseases. For this reason, we think the negative relationship between NC and LDL-C is due to the use of lipid-lowering drugs in most participants.

The key limitations of our study are that it is a cross-sectional study, valid standard methods such as BIA were not used to determine the body fat or lean mass and could not be evaluated, and the relationship was analyzed only in terms of routine blood parameters including LDL-C, TG, FBG, and HbA1c with NC measurement in this study. Although there are studies on the risk of NC in metabolic syndrome [25,28,29] and cardiovascular diseases [9,12,23,29], the number of studies on T2DM is limited. In addition to BMI and WHR, unlike other studies, the study's strength is that the relationship between WHtR, an important indicator of abdominal obesity, and NC was also evaluated.

## Conclusions

The study shows that NC measurement significantly correlated with other anthropometric measurements in both genders and can be a good indicator of the accumulation of upper subcutaneous adipose tissue in people living with diabetes. Also, the effect of higher NC, an indicator of upper subcutaneous adipose tissue, on glucose homeostasis in males with T2DM is due to the relationship between FBG and HbA1c.

Neck circumference is simple, unaffected by external factors, can be measured in a short time, and is an easy and reliable measurement method that can be used in both clinical and epidemiological studies. However, prospective, followed-up studies with larger samples are needed, categorized according to the effects of drugs on body weight to evaluate the relationship of NC with other anthropometric measurements and biochemical parameters in individuals with diabetes.
